# Ecological Niche Modeling of *Bacillus anthracis* on Three Continents: Evidence for Genetic-Ecological Divergence?

**DOI:** 10.1371/journal.pone.0072451

**Published:** 2013-08-19

**Authors:** Jocelyn C. Mullins, Giuliano Garofolo, Matthew Van Ert, Antonio Fasanella, Larisa Lukhnova, Martin E. Hugh-Jones, Jason K. Blackburn

**Affiliations:** 1 Spatial Epidemiology & Ecology Research Laboratory, Department of Geography, University of Florida, Gainesville, Florida, United States of America; 2 Istituto Zooprofilattico Sperimentale dell’Abruzzo e del Molise “G. Caporale”, Teramo, Italy; 3 Anthrax Reference Institute of Italy, Istituto Zooprofilattico Sperimentale della Puglia e della Basilicata, Foggia, Italy; 4 Emerging Pathogens Institute, University of Florida, Gainesville, Florida, United States of America; 5 Anthrax Laboratory, Kazakh Science Center for Quarantine and Zoonotic Diseases, Almaty, Kazakhstan; 6 Department of Environmental Sciences, School of the Coast and Environment, Louisiana State University, Baton Rouge, Louisiana, United States of America; Rockefeller University, United States of America

## Abstract

We modeled the ecological niche of a globally successful *Bacillus anthracis* sublineage in the United States, Italy and Kazakhstan to better understand the geographic distribution of anthrax and potential associations between regional populations and ecology. Country-specific ecological-niche models were developed and reciprocally transferred to the other countries to determine if pathogen presence could be accurately predicted on novel landscapes. Native models accurately predicted endemic areas within each country, but transferred models failed to predict known occurrences in the outside countries. While the effects of variable selection and limitations of the genetic data should be considered, results suggest differing ecological associations for the *B. anthracis* populations within each country and may reflect niche specialization within the sublineage. Our findings provide guidance for developing accurate ecological niche models for this pathogen; models should be developed regionally, on the native landscape, and with consideration to population genetics. Further genomic analysis will improve our understanding of the genetic-ecological dynamics of *B. anthracis* across these countries and may lead to more refined predictive models for surveillance and proactive vaccination programs. Further studies should evaluate the impact of variable selection of native and transferred models.

## Introduction

*Bacillus anthracis* is a soil-borne, spore forming bacteria and the causative agent of anthrax in wildlife, livestock and humans worldwide. Metabolically dormant *B. anthracis* spores can persist in landscapes with suitable soil and ecological characteristics for long periods of time [1] such that years may pass between outbreaks. More recent evidence suggests *B. anthracis* may have an active soil life cycle [2]. Despite a long recorded history of anthrax [3], the environmental and epidemiological catalysts for epizootics are poorly understood. Control of the disease in livestock and humans is best achieved through annual vaccination of livestock and adequate surveillance to identify outbreaks early in the epidemic course [4]. In wildlife populations, control is limited to active surveillance and proper disposal of carcasses [5]. The economic conditions in many anthrax endemic areas, however, combined with expansive rural geographies are such that early recognition of outbreaks and proactive distribution of vaccine is challenging, but could be facilitated by active, targeted efforts in areas of high likelihood of occurrence. Ecological niche models of *B. anthracis* identify geographic areas suitable for pathogen persistence, and these areas should be considered priorities for surveillance and vaccination. In particular, areas predicted to be supportive of the pathogen which also have a history of outbreak clusters should be targeted for active control [6].

Much of the earlier literature on anthrax ecology describes soil conditions that are favorable for *B. anthracis* persistence, including higher calcium levels and pH [7–9]. In addition to soil, there is evidence that endemic anthrax areas are associated with warmer temperatures, higher soil moisture content and topography [9,10]. These relationships between potential pathogen persistence and these environmental variables make ecological niche modeling valuable for predicting the spatial distribution of anthrax. Ecological niche models (ENMs) are frequently used to predict the potential distributions of species in ecologic and geographic space. A variety of correlative algorithms are available, all of which aim to identify non-random relationships between known location data for the species and environmental variables and then identify geographic areas of predicted presence. Models developed on a native landscape and determined to accurately predict the species’ presence in the native range can be projected, or transferred, onto novel geographical areas. Transferred models have been used to predict suitable areas for invasive species [11,12], to predict the effects of climate change on species’ range [13,14], and to test niche conservatism hypotheses [15,16]. Ecological niche modeling has been incorporated into phylogeographic studies by evaluating whether genetically defined sub-populations are associated with divergence in ecological niche and geographic distribution [17–21]. Combining ENM and phylogeography is particularly informative for studies of globally distributed pathogens which have intricate, often little understood, interactions with spatially heterogeneous abiotic and biotic factors which may be linked to genetic variation. From a methodological perspective, widely distributed species tend to result in less accurate ecological niche models [22], and models for such species can be improved by dividing the larger population into biologically meaningful sub-populations such as those defined by genetic analysis [17,23].

The phylogeography of *B. anthracis* has been described at the global level [24] and in multiple regions [7,25–27] using a combination of genetic markers including single nucleotide polymorphisms (SNPs) [24] and multiple locus variable number tandem repeats (MLVA) [24,28,29]. Although the geographic distribution of genetic lineages as defined by SNP and MLVA analysis exhibits heterogeneity, globally successful lineages can dominate strain collections in countries on separate continents. For example, the MLVA defined A1.a is widely distributed throughout North America and Eurasia [24]; this sublineage was further defined using SNP analysis into the trans-Eurasian ancestor (TEA) and the related Western North American (WNA) group [24].

The possibility that genetic variation in bacterial pathogens is associated with spatial and ecological divergence is supported by studies demonstrating unique epidemiologic characteristics or ecological affinities among genetic groups of pathogens within a geographic region [17,18,30]. In the case of *B. anthracis*, a study in Kruger National Park, South Africa [7] revealed intriguing genetic-ecological associations for the pathogen and a differential distribution of genotypes based on soil characteristics. In this study, Smith et al. [7] detected spatial and ecological differences between genotypes representing the evolutionarily distinct *B. anthracis* A and B branches within the relatively narrow geographic boundaries of Kruger National Park. More recently, work by Mullins et al. [21] suggested that *B. anthracis* genotypes belonging to A1.a sublineage in Kazakhstan were associated with a broader ecological space than the larger population containing multiple A cluster sublineages, further supporting the importance of genetic information in building relevant ENMs for the species.

In the countries of United States, Italy and Kazakhstan, strains belonging to the A1.a sublineage are ecologically established and dominate strain collections [24–26,28,29], providing a unique opportunity to study the dynamics of this successful group across diverse landscapes. In the United States, ecological niche modeling of anthrax outbreak locations predicted pathogen persistence primarily along a narrow corridor running from southwest Texas northward through the Dakotas [4]. In that landscape the A1.a sublineage (SNP group WNA) is dominant, although small areas appear to support other sublineages, including A3.b and A4. The genetic diversity of *B. anthracis* and geographic distribution of anthrax outbreaks in Italy was described by Fasanella et al. [26], Lista et al. [29] and Van Ert et al. [24], and confirmed by Garofolo et al. [31]. The dominant genotypes in Italy fell into the MLVA A1.a group and TEA SNP group. Isolates belonging to the A1.a/TEA sublineage also predominate in the southern portion of Kazakhstan [25], where the ecological niche and predicted geographic distribution has been modeled [21,32]. We developed ecological niche models of this globally successful *B. anthracis* sublineage in the United States, Italy and Kazakhstan. Models were reciprocally transferred to determine if pathogen presence could be accurately predicted on novel landscapes.

## Methods

### Anthrax occurrence data and environmental data

**Figure 1:**
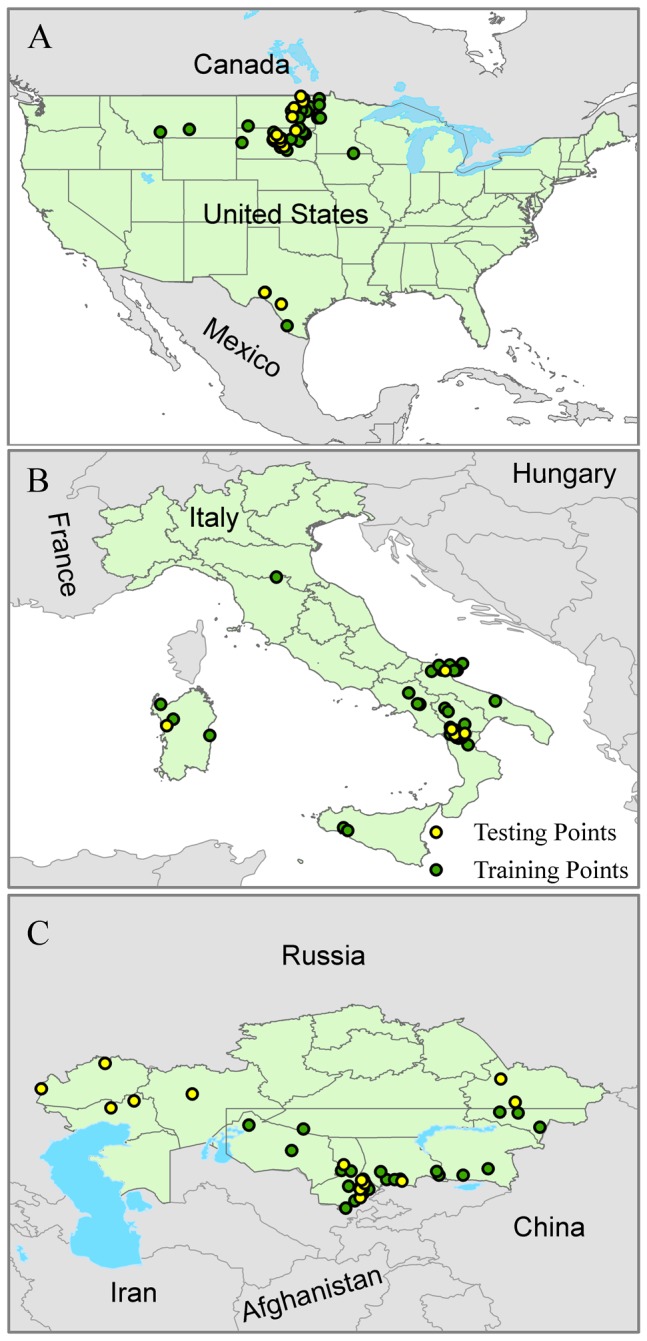
Geographic distribution of the training and testing points used for ecological niche model building and evaluation. Occurrences are shown for (A) the United States, (B) Italy and (C) Kazakhstan. Model training points are illustrated in green and independent data for model evaluation are yellow.

Geographic information system (GIS) databases of *B. anthracis* isolates belonging to the MLVA-defined A1.a sublineage and SNP defined TEA/WNA from the United States (U.S.), Italy, and Kazakhstan were used to support our analyses. All isolates were derived from collections previously genotyped using MLVA typing systems containing the set of eight markers (MLVA-8) described in Keim et al. [28] and SNP analysis [24]. The U.S. isolates were derived from Kenefic et al. [33], Blackburn et al. [4] and new strains from recent field collections in western Montana and Texas (Blackburn, unpublished data). The isolates from Italy were first reported in Fasanella et al. [26] and mapped for this study. Several isolates from Italy were mapped only to the nearest province and, therefore, we removed the isolates from the analysis. Kazakh data were derived from Mullins et al. [21]. Isolates were independently grouped into genetic lineages using the unweighted pair group method with arithmetic mean (UPGMA) cluster analysis or canSNP group assignments [24–26]. Remaining occurrence data were reduced to spatially unique points at an 8 km^2^ resolution to accommodate the spatial uncertainty of the data [13]. Each occurrence dataset was randomly divided into an 80% training set for model building and a 20% dataset for testing the native projection. Figure 1 shows the occurrence point distributions for each of the countries used in this analysis.

Grids representing six bioclimatic variables (Table 1) and altitude were downloaded from WorldClim (www.worldclim.org) and two satellite-derived environmental variables describing measures of vegetation were obtained from the Trypanosomiasis and Land Use in Africa (TALA) Research Group (Oxford, United Kingdom; Table 1) [34,35]. WorldClim bioclimatic grids (Bioclim) may be more biologically meaningful than annual mean, maximum and minimum values because the manipulation of monthly data results in variables that represent annual trends and seasonality as well as extremes in environmental conditions that limit a species’ range. All grids were resampled to 8 km^2^ and clipped to country boundaries using ArcView 3.3 with the GARP datasets extension (Environmental Systems Research institute, Redlands, California, USA). The eight variables in the environmental dataset were chosen based on parameters reported to reflect persistence of *B. anthracis* in soils and used in previous studies [4,13,32,36]. The background extent of each country was determined by political boundaries. These extents were chosen because reporting and control of anthrax is conducted within political boundaries.

**Table 1:** Environmental variables used to develop ecological niche models.

**Environmental Variable (unit)**	**Name**	**Source**
Elevation (m)	Altitude	WorldClim^†^
Annual Temperature Range (°C)	BIO7	WorldClim
Annual Mean Temperature (°C)	BIO1	WorldClim
Precipitation of Driest Month (mm)	BIO14	WorldClim
Precipitation of Wettest Month (mm)	BIO13	WorldClim
Annual Precipitation (mm)	BIO12	WorldClim
NDVI Amplitude (no units)	wd1014a1	TALA^‡^
Mean NDVI (no units)	wd1014a0	TALA

^†^ (www.worldclim.org) [35]

^‡^Trypanosomiasis and Land Use in Africa (TALA) Research Group (Oxford, United Kingdom) [57]

### Model development

This study employed the Genetic Algorithm for Rule-Set Prediction (GARP) [37] with a best subset procedure to perform the ecological niche modelling experiments [38]. Briefly, the GARP approach uses a two-step procedure in which sets of rules are developed iteratively to predict presence or absence in variable space using presence only input data and background data. These rule sets are combinations of if/then statements derived from either variable ranges or logistic regression functions. This process is a random walk and develops multiple models; the best subset procedure aids in the selection of optimal models based on user-defined thresholds of omission and commission. Optimal rule sets are then projected onto the landscape. Training data were input into GARP with a 50% training/50% testing internal data partition. For all experiments, we specified 200 models with a maximum of 1,000 iterations and a convergence limit of 0.01. The 10 best subset models were selected using a 10% hard omission threshold and a 50% commission threshold. These output models were imported into ArcMap 10 (Environmental Systems Research institute, Redlands, California, USA) and summated to generate a single cumulative raster file of model agreement for *B. anthracis* presence. Grid cell values thus ranged from 0 (all models predict absence) to 10 (all models predict presence). Native models were trained in each of the three countries, then rule sets were projected onto the two other landscapes (for example, the model trained in the U.S. was projected onto Kazakhstan and Italy). As described in Mullins, et al. [21], Kazakh models were trained with a subset of southern A1.a isolates and projected onto the entire Kazakh landscape, the U.S. and Italy.

### Model evaluation

Predictive performance of the best model subset was evaluated with an area under the curve (AUC) in a receiver operating characteristic (ROC) analysis using withheld independent test data for native models and all data for projected models. Values of AUC approaching one indicate a well-performing model while an AUC equal to 0.5 indicates the model performs no better than random and are tested statistically with a z-score (Z) and standard error (SE) estimates. AUCs were interpreted in conjunction with measures of omission and commission calculated using the summated 10 best models [39,40]. Total commission is the percent of pixels which are predicted as presence by areas of 10 model agreement. Average commission is the average area predicted as presence by all subset models. The greater the difference between the 2 measures of commission, the greater the spatial heterogeneity among the 10 best subset models [22]. Two measure of omission were also calculated. Total omission was calculated as the total number of test points falling into areas predicted as absence by all 10 models. Summed area omission (SAO) was calculated as the omission error of areas of 10 model agreement. Models with small SAO values are desirable because complete model agreement represents the most conservative threshold with which to predict areas of presence. Models which most robustly predict areas with a high likelihood of pathogen persistence facilitate implementation of cost effective, focused public health measures such as surveillance and pre-emptive vaccination.

## Results

**Figure 2:**
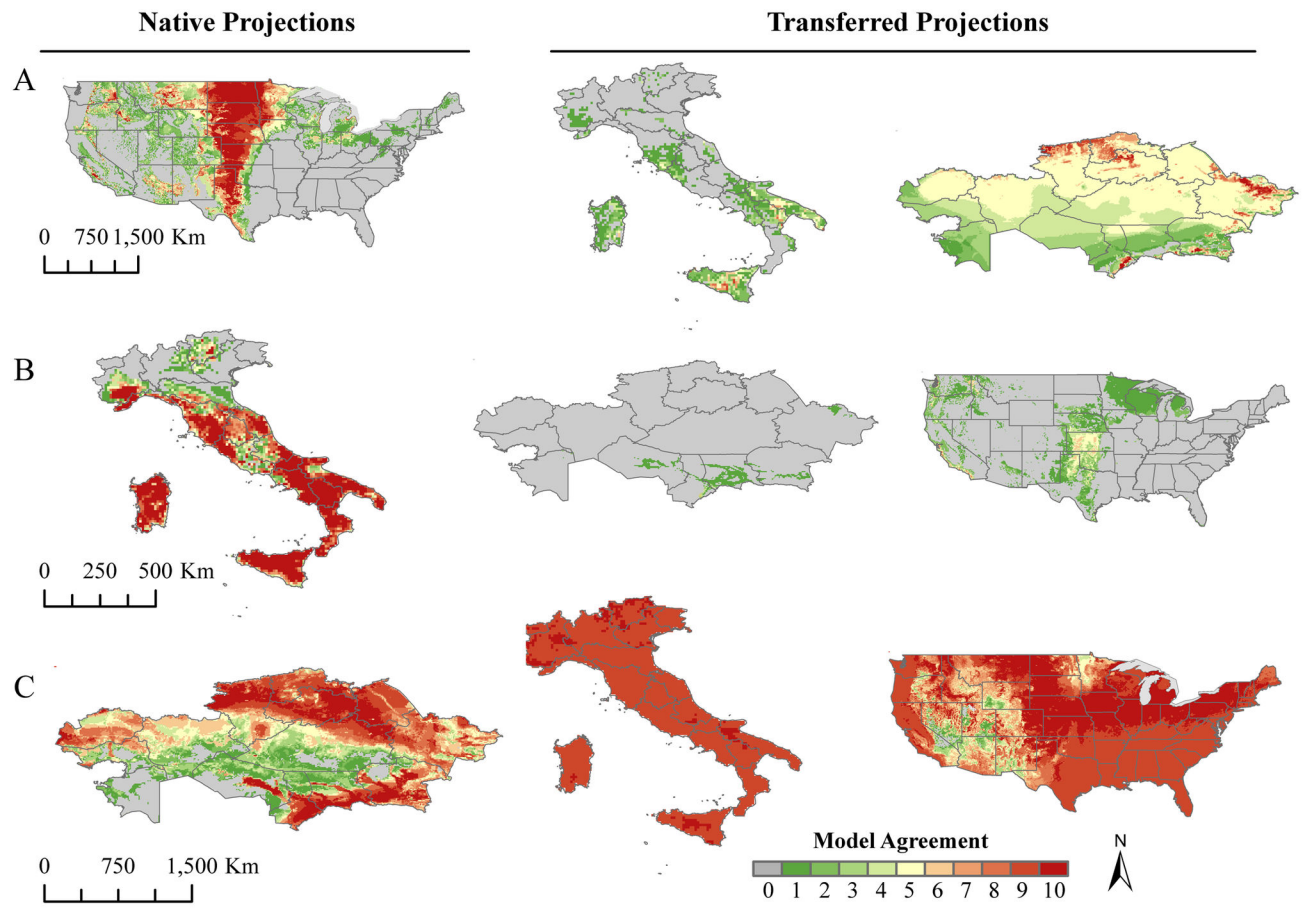
Predicted distribution of *Bacillus anthracis* by native and transferred projections. Native models were built for (A) the United States, (B) Italy and (C) Kazakhstan. Color ramp indicates the level of model agreement from zero (no models predict presence) to ten (all models in the best subset predict presence).

All experiments reached convergence of accuracy prior to the maximum 1,000 iterations. The AUC scores for native projections all performed significantly better than random, and native models each had zero total omission and low average omission (Table 2). The native U.S. model predicted *B. anthracis* distributed in a north–south corridor in the center of the country (Figure 2). This band widens as it moves from southwest Texas northward into South and North Dakota and eastern Montana. Areas of the interior northwest are also predicted. The native Italian model predicted large sections of the southeastern mainland as well as the islands of Sardinia and Sicily. Areas of high likelihood also include coastal regions in central Italy and two relatively isolated regions of the northeastern and central north portions of the country. The Italian model accurately predicted areas of provinces in the northeast where A1.a strains have been documented, but were excluded from the analysis because of imprecise GIS data. In Kazakhstan, the native projection strongly predicted presence along the mountainous region of the southern portion of the country and a broad area in the north, while predicting the interior of the country with low model agreement.

**Table 2:** Sample sizes and accuracy metrics for all native models and projections.

Training Landscape	United States			Italy			Kazakhstan^†^			
Projection	Native	IT	KZ	Native	KZ	US	Native	KZ	IT	US
Training	48	-	-	28	-	-	24	-	-	-
Testing	12	35	39	7	39	60	8	15	35	60
AUC	**0.93**	0.51	0.48	**0.84**	0.56	0.43	**0.90**	0.71	0.45	0.48
SE	0.05	0.05	0.05	0.09	0.05	0.04	0.09	0.08	0.05	0.04
Z	5.84^‡^	7.72^‡^	5.99^‡^	3.86^‡^	97.91^‡^	16.42^‡^	3.13^‡^	4.52^‡^	15.93^‡^	7.71^‡^
Total Omission	0	62.1	13.2	0	86.8	96.4	0	0	0	0
Average Omission	6.7	71	51	1.4	74.8	82.2	0	19.2	10	17.7
Total Commission	8.38	0	0.84	27.86	0	0	19.77	13.17	9.04	28.67
Average Commission	22.88	7.71	43.54	50.21	0.05	3.17	46.3	54.28	90.9	83.35
SAO	16.67	-	89.47	14.29	-	100	0	46.15	100	62.50

Native projection are under the curve (AUC) scores are shown in bold for comparison. SE = standard error, Z = z-score, SAO = summed area omission.

^†^ Native training and testing are for southern training area only

^‡^ statistically significant value

Models projected to novel landscapes performed poorly. Transferred models which performed better than random were poor, and some transferred models were more dispersed than random; measures of omission and commission were consistent with under-prediction by the Italian and U.S. models and over-prediction by the Kazakh model (Figure 2, Table 2). The native U.S. model when transferred to Italy predicted similar geographic areas of Italy as the native Italian model, but with very low model agreement. The transferred model failed to predict the area in southern Basilicata which has experienced anthrax outbreaks and gave low agreement for known endemic areas. The native U.S. model transferred to Kazakhstan broadly predicted, with low model agreement, almost the entire landscape. Small areas in the southern and northern regions were predicted with higher model agreement. These areas were also predicted by the native Kazakh model, whereas in the northeast corner of the country, the U.S. and Kazakh models were very different.

The model trained in Italy transferred to Kazakhstan predicted a highly endemic area of southern Kazakhstan, although with very low model agreement. Projected onto the U.S., the model trained in Italy did highlight some areas predicted by the native trained model, namely western Texas, western Oklahoma and Colorado, but again with low model agreement. When transferred, the models trained in Kazakhstan over-predicted all of Italy and the majority of the landscape in the U.S.

## Discussion

Evidence increasingly suggests phylogenetic analysis provides a meaningful way to subdivide *B. anthracis*, and other species, for more accurate niche modeling [7,17,19,21,23]. Furthermore, an ability to apply models developed in a landscape with known anthrax locations to one in which anthrax is not reported or is poorly recognized would be of great value in predicting potential areas of emerging disease. This study tested whether ecological niche models of the *B. anthracis* MLVA-8 defined A1.a sublineage can be used to predict the distribution of the same sublineage on novel landscapes. Our native models reasonably predicted the occurrence points in our experiments, and furthermore the native Italian model predicted a region in Italy where the A1.a sublineage has been isolated, but due to lack of spatial data was not included in the occurrence dataset. Transferred models, however, failed to accurately predict documented anthrax occurrence points and tended to over-predict or under-predict presence on the non-native landscapes. Where transferred models were successful in predicting areas of known persistence, model agreement tended to be low. Both over-prediction and under-prediction of transferred models are considered failures, although these failures have different practical consequences for public health applications. When areas suitable for pathogen presence are not predicted by a model, the failure to incorporate this area into surveillance programs will result in cases of disease being overlooked. Over-prediction by a model, on the other hand, hinders epidemiologic investigations of cases by misallocating resources to monitor regions that are erroneously predicted to support the pathogen.

The failure of transferred models to accurately and consistently predict known occurrences presents an interpretive challenge. The lack of transferability may have resulted from methodological shortcomings of transferring models, or could reflect genetic-ecological divergence of the pathogen [15,17,19,41,42]. Evidence supporting genetic-ecological divergence was demonstrated in Kruger National Park, South Africa, where the A lineage had broader ecological tolerances than the B lineage within the park [7]. Our results suggest that this ecological divergence in *B. anthracis* strains may also occur in a widely distributed sublineage. It is possible that successful genetic groups with broader tolerances are more likely to become established across ecological extremes, and the local population would then differentiate to develop a unique genetic signature associated with the local ecology [43]. In contrast, *B. anthracis* lineages with more limited tolerances, such as the B lineage and, although its ecological associations have not been characterized, the geographically limited C lineage, would be restricted ecologically and geographically. This process of regional-scale differentiation leading to spatially structured genetic variation has been described among other introduced species or pathogens [15,17,18,33,44]. That Italian and U.S. models under predicted pathogen occurrence in Kazakhstan, whereas Kazakh models overpredicted a large extent of Italy and the U.S., may indicate that Kazakh strains have adapted to a broader ecological envelope. Although more complete genomic data are required to substantiate this hypothesis, this may reflect an earlier introduction of the sublineage into Kazakhstan than in Italy or North America.

Evidence for genetic differentiation within the MLVA-8 defined A1.a sublineage includes the separation of this sublineage into distinct TEA and WNA SNP groups [24]. More recent SNP analysis of the WNA lineage [33] suggests a significant evolutionary divergence between the North American (U.S.) and Eurasian (Italy and Kazakhstan) strains. The evolutionary divergence between Eurasian strains in Kazakhstan and Italy is not as well defined. However, a 15-marker VNTR analysis of the Eurasian group reported by Van Ert et al. [24] suggests a high level of genetic diversity exists within the ‘TEA’ SNP group (A. Br.008/009). More recent VNTR based analyses based on 25 markers indicates that the Kazakh ‘A1.a’ strains, as previously defined using the 8-marker system, exhibit a considerable degree of genetic divergence (Sytnik, unpublished data) from European strains. Although this VNTR based diversity is intriguing, more comprehensive analysis is required to more accurately measure the evolutionary relationships between these populations, particularly considering that discovery bias inherently limits resolution in ‘canonical’ SNP data [45]. There is clearly a need to genotype existing collections of *B anthracis* isolates with the highest resolution systems available, such as the Lista 25 marker system [29] or the 31 marker system [46]. Such an effort will enhance our understanding of the phylogenetics and the ecology of the pathogen. Existing niche models should then be reconstructed according to emerging phylogenetic evidence. Despite the limitations of the genetic data used in the present analysis, the finding that native models were successful while transferred models failed to predict anthrax occurrence points suggests that niche specialization may have occurred within this broadly distributed sublineage and this suggestion of genetic-ecological associations warrants additional investigation.

From a modeling perspective, however, the effect of variable selection [41,42,47,48] and background [49–51] on transferred projections must be considered as potential methodological limitations when interpreting the results of this study. The variable set used in the current experiments was chosen based on variables considered to be limiting factors in the persistence of *B. anthracis* based on previous niche modeling efforts performed within single landscapes [4,21,32]. In studies exploring the effects of variable selection on transferred ecological niche modeling experiments, changes in the dimensionality [41,52] and source [41] of environmental datasets resulted in different geographic predictions, and these difference were more pronounced in the novel landscapes than in the native ones. Variable selection can limit transferability because limiting factors for the species vary geographically [42,49], and, in addition, interactions among ecological variables may differ across landscapes, which would alter the relationship of more distal variables with the pathogen [51]. Ecological variables limiting the spatial distribution of *B. anthracis* may also vary with genetic lineage, reflecting niche specialization suggested by the work of Smith et al. [7] in Kruger National Park, South Africa. The impact of variable selection, as well as that of different techniques for selection of environmental variable sets, on these results will be evaluated in additional experiments.

A second methodological consideration is that of the extent used for background. We have defined the background in this study according to political boundaries instead of using other suggested techniques such as minimum convex polygons [42], global ranges [53], or the accessible extent [50,51] because *B. anthracis* dispersal to novel areas is primarily anthropogenic and therefore not constrained by natural physical boundaries or biological limits to movement. Therefore the potential area of dispersal, as currently understood, is limited by control and prevention measures which follow political boundaries. Similarly, the nature of surveillance for, diagnosis of and reporting of anthrax outbreaks is dependent on policies which fall within such limits. Despite this practical consideration, however, we recognize that within the political boundaries used for defining the extent for these models, considering the large geographic extents and latitudinal differences between the countries, the ranges of environmental variables is likely differ between landscapes. Hence, concerns about extrapolation are significant and should be addressed in additional experiments.

While we suggest the results presented here may reflect niche differentiation within this sublineage of *B. anthracis*, while considering the potential effects of methodological problems, it is important to also consider the source of isolate/occurrence data and the effects of control efforts on the disease in each country [4]. It is possible that the broad native predictions within Kazakhstan reflect an insufficient capacity to maintain widespread vaccination to reduce the overall burden of anthrax [54]. In the case of the U.S., models likely reflect areas where the disease persists after decades of widespread vaccination efforts have resulted in a clear reduction in overall outbreak numbers and an accompanying contraction in the spatial extent of disease [55]. Differences in historical control, alone or in synergy with genetic and ecological factors, could explain some portion of the over- and under- prediction observed in the transference of models.

In this study we have used measures of omission, commission and AUC as accuracy metrics to evaluate native models and their transferability. The AUC measure has limitations when evaluating ecological niche models [39,40]. Two described limitations are that AUC is influenced by the geographic extent of the landscape being studied and the AUC uses the entire ROC plot which would render comparisons between modeling platforms unreliable. Here, however, we evaluate models over the same extent (ie, a native U.S. model and a projection transferred to the U.S.) and all using the GARP modeling platform. The optimum weighting of omission and commission, although equally weighted in the AUC, varies depending on the purpose of the experiment. In this study ENM was used to predict the potential presence of a pathogen and infer subsequent disease risk to inform public health policy. Errors of commission, or over-prediction, will result in excess expenditures for surveillance and interventions and mislead trace-back efforts, whereas errors of omission, or under-prediction, could result in sustained transmission [56]. Viewed in this context, optimal weighting of omission and commission will depend on the relative costs of surveillance and disease. The summed area omission (SAO) allows for refinement of models with the goal being that areas of total model agreement can be used as a conservative threshold for predicted presence. By calculating omission only in areas of complete model agreement, this conservative evaluation of model performance maximizes predictive value without incorporating potentially large geographic areas of low model agreement and therefore lower risk. Expensive surveillance and prevention programs can then be effectively targeted to highest risk areas.

Previous ecologic niche models described conditions favorable for *B. anthracis* outbreaks based on collections of isolates from multiple genetic lineages that were likely biased towards a dominant subset of genotypes [4,32] and the potential for using genetic analysis to improve models was subsequently demonstrated [21]. Our findings suggest genetic-ecological divergence exists among geographically dispersed populations of *B. anthracis* from the MLVA-8 defined A1.a sublineage. Some caveats apply, however. More comprehensive and higher resolution genomic data is required to better characterize the genetic differences between these populations. In addition, the methodological problems discussed here must be explored in order to refine native models and to evaluate whether variable selection methods will enhance the transferability of models. Until our understanding of the genetic-ecological dynamics of *B anthracis* is better developed, we suggest that *B. anthracis* is best modeled on a country or regional level and with consideration of the genetic diversity of the population. Moving forward, understanding *B. anthracis* genetic-ecological associations on the landscape will result in construction of ecological niche models that are sensitive to genotype and region and are more successful in predicting outbreaks.
